# From clinical phenotypes to molecular precision: multimodal biomarkers for progressive supranuclear palsy

**DOI:** 10.3389/fnins.2026.1893149

**Published:** 2026-07-27

**Authors:** Glenn A. Harris, Lymor Barnhard, Jennifer Brummet, Kristophe Diaz

**Affiliations:** 1 Rainwater Charitable Foundation, Fort Worth, TX, United States; 2 CurePSP Inc., New York, NY, United States

**Keywords:** biomarkers, neurodegeneration, neuroimaging, PSP, tau, tauopathy

## Abstract

Progressive Supranuclear Palsy (PSP) is the most prevalent primary 4R-tauopathy, characterized by the pathogenic accumulation of misfolded tau protein within neurons and glial cells. Historically, clinical diagnosis relied upon the identification of Richardson’s Syndrome, however, the recognition of diverse clinical phenotypes that overlap with Parkinson’s disease, corticobasal syndrome, and frontotemporal dementia has complicated the diagnostic landscape and hindered the success of developing therapeutic interventions. As the field transitions toward a precision medicine paradigm, there is a growing need for validated biomarkers that can provide molecular specificity, facilitate early diagnosis, and accurately track disease progression. This paper reviews the recent advancements in neuroimaging and fluid-based biomarkers, assessing their potential to delineate PSP from similar neurodegenerative conditions and unlock the 4R-tau therapeutic pipeline. In the domain of neuroimaging, while structural magnetic resonance imaging (MRI) and the Magnetic Resonance Parkinsonism Index (MRPI) continue to provide measures of subcortical atrophy, the emergence of second-generation tau-selective positron emission tomography (PET) radioligands represents a transformative shift. New tau PET tracers offer the ability to visualize tau pathology *in vivo*, providing a more direct assessment of the underlying proteinopathy than traditional volumetric measures. These advancements are complemented by significant progress in fluid biomarkers. Plasma phosphorylated tau at residue 217 (p-tau217) has gained prominence as a robust marker for Alzheimer’s disease, and its primary utility in PSP research currently serves as a critical negative signature to exclude amyloid-associated co-pathology. In contrast, novel assays targeting microtubule-binding region tau fragments show burgeoning potential for the specific identification of 4R-tau isoforms. Furthermore, neurofilament light chain (NfL) has been firmly established as a sensitive, albeit non-specific, indicator of neuroaxonal injury and clinical severity. Additional advancements with digital health approaches and electrophysiological assessments add to the opportunities for improved objective measures. This review concludes that the shift from clinical-only diagnostic criteria to a biomarker-enabled molecular framework is the necessary catalyst for developing effective disease-modifying therapies for PSP and related 4R-tauopathies. The synthesis of these multimodal biomarkers into a unified framework will be essential to improve participant stratification, enable the use of adaptive trial models, and provide supportive evidence of target engagement for future clinical trials.

## Introduction

Progressive Supranuclear Palsy (PSP), first characterized as a distinct clinicopathological entity by Steele, Richardson, and Olszewski in 1963 (Steele et al., 1964), represents the most common atypical parkinsonian syndrome with a reported pooled incidence of approximately 7 per 100,000 (Barer et al., 2022; Swallow et al., 2022; Lyons et al., 2023). Historically, the disease was identified by vertical supranuclear gaze palsy, axial rigidity, pseudobulbar palsy, and early postural instability leading to falls. This classic presentation is now designated as PSP-Richardson’s Syndrome (PSP-RS) (Williams et al., 2005). However, clinico-pathological studies over the last two decades have revealed that PSP-RS accounts for only a portion of cases, with a significant number of patients presenting with diverse phenotypes mimicking Parkinson’s disease (PD), corticobasal syndrome (CBS), or frontotemporal dementia (FTD) (Williams et al., 2005; Höglinger et al., 2017). From the latest Movement Disorder Society Criteria of clinical variants of PSP, there are a total of 10 recognized variants of PSP clinical presentations, not including other diseases that may clinically resemble PSP (Table 1; Höglinger et al., 2017; Krzosek et al., 2022).

**Table 1:** Classification of PSP variants.

Variant	Abbreviation	Primary clinical features and key diagnostic indicators
Cerebellar	PSP-C	A rare presentation where prominent cerebellar ataxia and limb discoordination are the leading features. This variant often requires differentiation from Multiple System Atrophy (MSA-C).
Corticobasal syndrome	PSP-CBS	Defined by asymmetric cortical signs, including limb apraxia, cortical sensory loss, or alien limb phenomenon. It is often clinically indistinguishable from Corticobasal Degeneration (CBD) until eye signs appear.
Frontal	PSP-F	Presents with early behavioral changes (apathy, impulsivity) and executive dysfunction similar to behavioral variant Frontotemporal Dementia (bvFTD). Motor signs may not emerge until years after cognitive onset.
Ocular motor	PSP-OM	Diagnosed when vertical supranuclear gaze palsy or significant saccadic slowing is the predominant feature without early falls or parkinsonism. It captures the hallmark ocular signs in isolation.
Parkinsonism	PSP-P	Mimics Parkinson’s disease with asymmetric limb bradykinesia, rigidity, and a transient response to levodopa. Hallmark PSP ocular and postural signs are often delayed by several years.
Postural instability	PSP-PI	Focuses on cases where unprovoked falls and balance failure are the primary symptoms early in the disease course, prior to the development of vertical gaze palsy.
Primary lateral sclerosis	PSP-PLS	A rare mimic of motor neuron disease, presenting with isolated upper motor neuron signs such as spasticity and hyperreflexia. Post-mortem tau pathology confirms the PSP diagnosis despite the ALS-like presentation.
Pure gait freezing	PSP-PGF	Characterized by isolated “start hesitation” and freezing of gait without early rigidity, tremor, or cognitive decline. This was previously referred to as Pure Akinesia with Gait Freezing (PAGF).
Richardson’s syndrome	PSP-RS	The classic phenotype featuring early postural instability (backward falls) and vertical supranuclear gaze palsy. It typically presents with a rapidly progressive, symmetric akinetic-rigid syndrome.
Speech and language	PSP-SL	Primary symptoms involve non-fluent/agrammatic primary progressive aphasia or progressive apraxia of speech. It is distinguished from other aphasias by the eventual development of PSP-related motor features.

The pathophysiological unification of these varied clinical presentations lies in their shared molecular pathology: the aggregation of microtubule-associated protein tau (MAPT). The tau protein exhibits high heterogeneity, with structural diversity resulting from the alternative splicing of its encoding gene, *MAPT*, located on human chromosome 17 (Frost, 2023). Within the human central nervous system, this splicing process generates six major tau isoforms. The six isoforms are classified based on two key structural features. The first is the number of N-terminal inserts, generated by the alternative splicing of exons 2 and 3. These inserts, located at the amino-terminal domain, are categorized as 0 N, 1 N, or 2 N (containing 0, 1, or 2 inserts of 29 amino acids, respectively) and modulate tau’s interaction with the plasma membrane and other proteins. The second feature is the number of microtubule-binding repeats in the C-terminal domain, determined by the inclusion or exclusion of exon 10 within the constitutive repeat region. Consequently, 3R-tau isoforms exclude exon 10 and contain three C-terminal repeats, while 4R-tau isoforms include exon 10 and contain four repeats. The combination of these N- and R-region features (e.g., 0N3R, 2N4R) results in the complete set of six major isoforms. In the healthy adult human brain, alternative splicing of exon 10 of the *MAPT* gene results in roughly equal ratios of tau isoforms containing three (3R) or four (4R) microtubule-binding repeats. PSP is characterized by a specific imbalance in this splicing, leading to the overproduction and aggregation of 4R tau (Williams and Lees, 2009). This distinguishes PSP from Alzheimer’s disease (AD), which involves paired helical filaments composed of both 3R and 4R tau, and Pick’s disease, a predominant 3R tauopathy.

The most specific microscopic hallmark of PSP is the tufted astrocyte. Unlike the astrocytic plaques found in Corticobasal Degeneration (CBD) which consist of tau-positive distal processes, tufted astrocytes exhibit dense, radially arranged tau filaments in the proximal, peri-nuclear processes of the cell resembling a star-like appearance centered around the nucleus (Takahashi et al., 2002). These inclusions are typically found in the motor cortex, striatum, and various brainstem nuclei. In diagnostic practice, silver stains or immunohistochemistry for phosphorylated tau are essential to visualize these signatures. The presence of at least one tufted astrocyte in a relevant region is now a core requirement for the “definite” neuropathological diagnosis under the Rainwater Charitable Foundation criteria (Roemer et al., 2022). Neuronal inclusions in PSP typically manifest as globose neurofibrillary tangles (NFTs) which are dense, rounded masses of tau (Yoshida, 2014). Staging is split across six steps determined by the spread of neuronal tau from its origins in the globus pallidus, subthalamic nucleus and substantia nigra through other brain regions along with the accumulation of astroglial and oligodendroglial tau pathologies originating in the striatum and globus pallidus, respectively (Kovacs et al., 2020). The hierarchical progression correlates with the clinical shift from motor symptoms to cognitive decline.

The integration of cryo-electron microscopy (cryo-EM) alongside neuropathology has fundamentally redefined our understanding of tauopathies, shifting the diagnostic paradigm from the cellular level to the atomic level (Fitzpatrick et al., 2017). While light microscopy identifies the “tufted astrocyte” as a hallmark of PSP, cryo-EM has revealed that the underlying molecular architecture of the tau filament itself is what truly distinguishes PSP from clinically similar 4R tauopathies. Tau filaments in PSP possess a unique, three-layered fold (Shi et al., 2021). The CBD core is larger and more complex, spanning a four-layer fold (Zhang et al., 2020). This structural divergence explains why certain PET ligands (e.g., ^18^F-flortaucipir) exhibit high affinity for the tau folds in AD but fail to bind effectively to the specific binding pockets of the PSP fold (Malpetti et al., 2022; Satoh et al., 2024).

**Figure 1:**
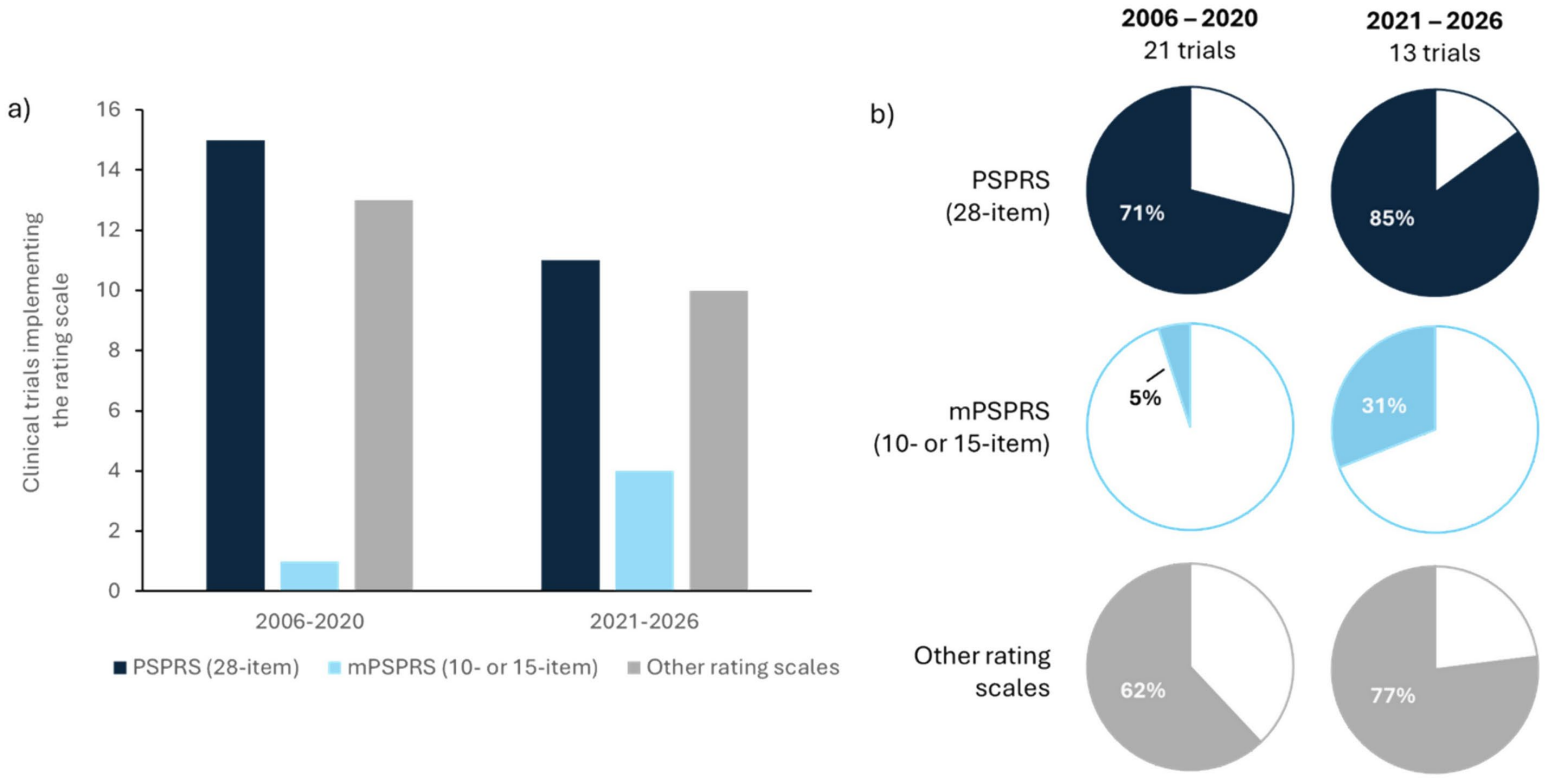
**(a)** The absolute number and **(b)** the relative percentage of clinical trials implementing PSPRS or other rating scales in the reported period.

Despite the growing understanding of tau pathology for patients, definitive antemortem diagnosis of PSP remains elusive. The current diagnostic criteria used to categorize a patient into a specific PSP phenotype is the Movement Disorder Society (MDS-PSP) criteria which emphasizes early detection and phenotypic diversity (Höglinger et al., 2017). The MDS-PSP criteria categorize patients into three levels of diagnostic certainty, Probable, Possible, and Suggestive of PSP, based on functional domains. Classification is based on core clinical features across the four domains of ocular motor dysfunction, postural instability, akinesia, and cognitive dysfunction, brain imaging, and clinical clues. MDS-PSP’s categorical structure is important for establishing a baseline and inclusion classification for a potential entry point for a clinical trial. In contrast, the PSP rating scale (PSPRS) is more of a metric of the disease burden (Golbe and Ohman-Strickland, 2007). That is, it is not used as a diagnostic instrument, but as a tool to measure progression and severity. It is scored based on the aggregate score of 28 items with each item being scored on either a 0-to-2 or 0-to-4 scale and summed to a maximum of 100. The PSPRS can be administered serially upon repeated clinical assessments which allow it to serve as a primary or secondary endpoint for outcome measurements in a clinical trial. While the PSPRS is a clinical staple, its reliance on specialized exam-based items may introduce inter-evaluator variability, and its utility may be limited by redundant item weighting and a lack of sensitivity to change over typical 12-to-18-month study durations (Grötsch et al., 2021). Consequently, researchers are exploring streamlined versions or composite endpoints to better reflect real-world functional decline and enhance statistical power. These newly proposed rating scales which use just 10 or 15 items instead of 28 are referred to as modified PSPRS (mPSPRS) which are gradually being implemented to accompany recent clinical trials (Piot et al., 2020; Grötsch et al., 2021; Wills et al., 2022; Gewily et al., 2024; Dam et al., 2025). Altogether the PSPRS remains the most used rating scale to support clinical outcome measure in PSP trials, but there is modest growth in running a mPSPRS alongside it in recent studies (Figure 1a). In the current period (2021-2026), 85% of the initiated clinical trials are utilizing the PSPRS while 31% are using a mPSPRS scale alongside it (Figure 1b).

This review aims to address a shift from the reliance on clinical rating scales alone as outcome measures toward a molecular precision framework, demonstrating how a new era of biomarkers could provide the solution. From an analysis of the initiated 34 initiated registered interventional clinical trials for PSP since 2006 (Supplementary Table S1), we discuss how fluid markers are enabling specificity in differentiating PSP from overlapping syndromes. Also, the paper will examine the crucial role of positron emission tomography (PET) and other neuroimaging approaches to help reliably stage disease severity *in vivo*. Finally, we explore how digital health technologies offer insight into disease progression. Ultimately, we argue that these biomarker-enabled strategies may unlock the tau-targeted and PSP therapeutic pipeline by enabling adaptive platform trials, improving patient selection, and providing biomarkers for target engagement to support the assessments made from clinical rating scales (Feldman et al., 2024; Harris et al., 2025).

## Overview of biomarkers supporting PSP clinical trials

### Neuroimaging-based biomarkers

Neuroimaging approaches like PET and magnetic resonance imaging (MRI) have been used to support PSP clinical trials since the initial reporting window of this study in 2006. Their use to support differentiation of clinical phenotype and understanding of the underlying tau pathology is integral to advancing clinical efforts (Table 2).

**Table 2:** Neuroimaging biomarkers in PSP including approaches, biological targets, and clinical utility to support clinical trials.

Modality	Specific approach	Biological target	Primary clinical utility	Outcome measure in PSP clinical trial (Clinicaltrials.gov registration number)
MRI	Structural	Macroscopic anatomy	Differential diagnosis; baseline screening	NCT02133846
	Volumetric	Regional brain volume	Progression tracking; clinical trial endpoint	NCT01110720; NCT02422485; NCT02460731; NCT04734379; NCT06162013; NCT07264283; NCT07217665; NCT07498426
	Diffusion	White matter integrity	Early detection; patient stratification	NCT02460731; NCT04734379
	QSM	Iron deposition	Progression tracking; tissue characterization	NCT04184063
	Neuromelanin	Pigmented nuclei	Differential diagnosis (vs. PD)	
	Functional	Network connectivity	Phenotypic biotyping; functional staging	NCT02422485; NCT02460731
PET	Tau	Tau aggregates	Differential diagnosis; progression tracking	
	FDG	Glucose metabolism	Diagnostic support; functional staging	NCT06162013; NCT04184063
	SV2A	Synaptic density	Diagnostic support progression tracking	
	TSPO	Microglial activation	Prognostic staging; tracking inflammation	

**Figure 2:**
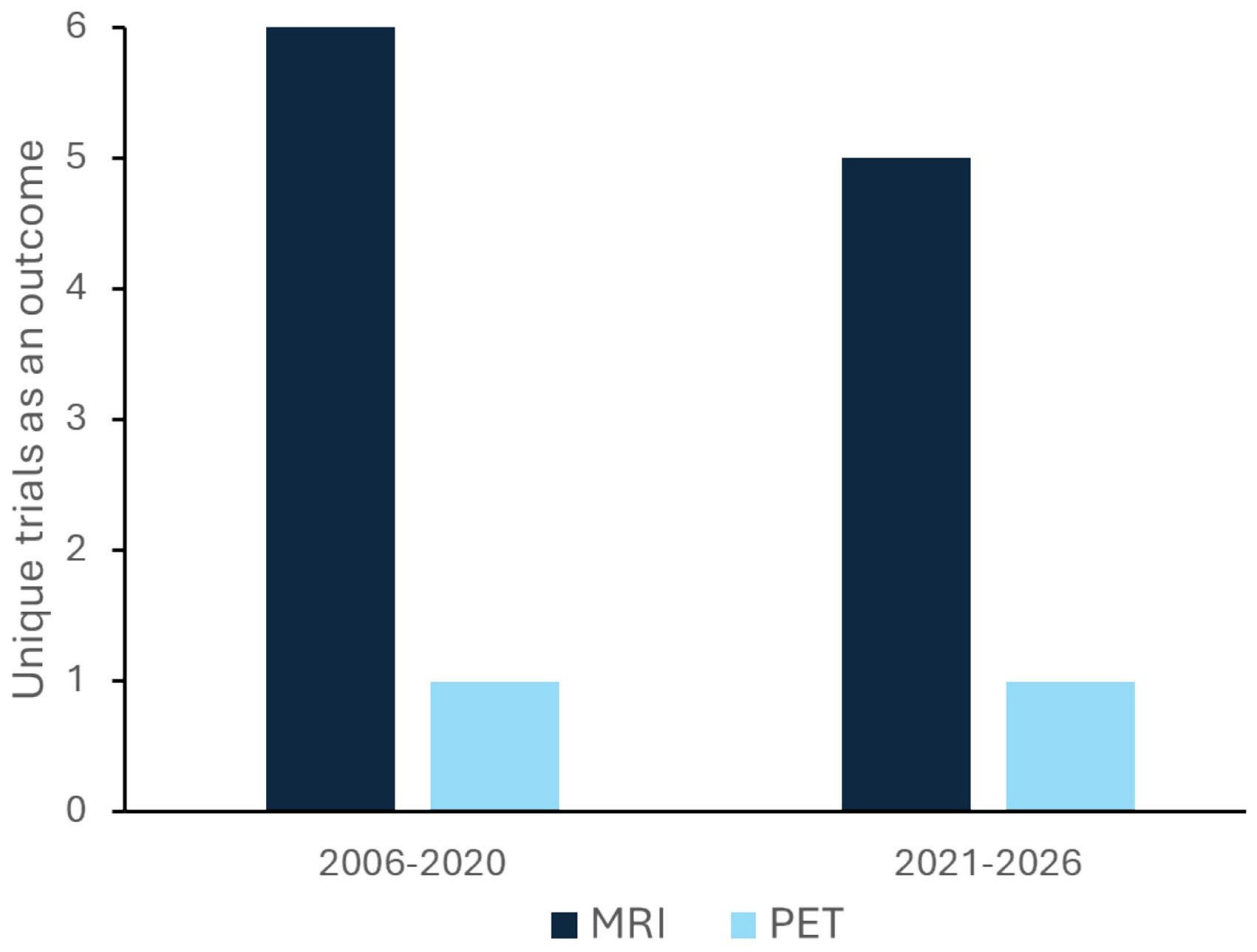
Neuroimaging approaches supporting PSP interventional clinical trials initiated during the referred time interval.

MRI is a cornerstone in the evaluation of PSP, providing critical insights into the macroscopic structural changes that characterize the disease. As a measure of downstream neurodegeneration, MRI quantifies the secondary neurodegeneration and atrophy resulting from neuronal loss and gliosis. These morphological changes serve as robust, non-invasive proxies for disease progression, and subsequently, MRI has been included as an outcome measure in 11 different interventional clinical trials for PSP (Figure 2).

#### Structural and planimetric assessment

Conventional structural MRI using high-resolution T1-weighted sequences has long been used to characterize the neuroanatomical signature of PSP. Qualitative assessment often, but not always, reveals selective midbrain atrophy with relative pontine preservation, classically described as the “hummingbird” or “morning glory” sign, while planimetric measurements enable objective characterization of this pattern (Mueller et al., 2018; Virhammar et al., 2022; Anyfantakis et al., 2025). Common metrics include midbrain area and the midbrain-to-pons ratio. More sophisticated composite indices such as the Magnetic Resonance Parkinsonism Index (MRPI) – defined as the (pons area/midbrain area) × (middle cerebellar peduncle width/superior cerebellar peduncle width) - and MRPI 2.0, which incorporates third ventricle width, have improved diagnostic sensitivity for differentiating PSP-RS from the PSP-P phenotype and other parkinsonian disorders (Quattrone et al., 2022). Longitudinal evidence indicates that MRPI 2.0 exhibits one of the highest annualized percentage changes among MRI-derived measures, although substantial inter-individual variability has been reported, which may limit its utility as a standalone progression endpoint (Quattrone et al., 2024). More recently, Quattrone and colleagues proposed the Dual-Line Midbrain PSP Index (DMPI), a simplified linear MRI biomarker that estimates midbrain atrophy using two easily acquired sagittal measurements. Early validation demonstrated diagnostic accuracy comparable to more complex planimetric indices while substantially simplifying image acquisition and analysis, highlighting its potential for broader implementation in routine clinical practice and multicenter studies (Quattrone et al., 2026).

#### Volumetric MRI

Volumetric MRI applies automated or semi-automated segmentation algorithms to derive quantitative measures of regional brain volume and longitudinal atrophy rates. Studies consistently demonstrate progressive volume loss in PSP across the midbrain, whole brainstem, superior cerebellar peduncle-associated regions, basal ganglia, and frontal cortical areas (Kumar et al., 2025). Importantly, regional atrophy rates can be detected over relatively short intervals (6-12 months), making volumetric MRI one of the most promising MRI-based progression biomarkers for this population. Volumetric measures correlate with clinical scales, such as the PSPRS, and are increasingly utilized as secondary or exploratory endpoints in interventional trials (Boxer et al., 2017). Notably, a multicenter study highlighted that while midbrain atrophy is a highly prevalent feature, regional atrophy patterns diverge across PSP clinical variants (Kumar et al., 2025). Their modeling suggests that combining targeted volumetric measures with the PSPRS yields greater statistical power than either measure alone, advocating for multimodal outcome strategies in future trials.

#### Diffusion-weighted imaging

Diffusion-weighted MRI has been used to study white matter damage longitudinally and microscopic changes in PSP over time. By quantifying the directionality of the microscopic movement of water molecules within brain tissue, a technique known as Diffusion Tensor Imaging (DTI), researchers can identify subtle damage to axonal tracts that may precede macroscopic atrophy on standard structural scans. Longitudinal diffusion abnormalities have been reported in PSP within the superior cerebellar peduncle, brainstem tracts, corpus callosum, and frontal white matter networks, with changes in fractional anisotropy and mean diffusivity correlating with disease severity and progression (Spotorno et al., 2019). These findings suggest that diffusion MRI may offer enhanced sensitivity for early progression tracking and staging. Recent frameworks using combined Region-of-Interest (ROI) and Tract-of-Interest (TOI) approaches have successfully mapped PSP stages *in vivo* to assist in patient stratification (Bârlescu et al., 2025). By applying these harmonized diffusion pipelines across multi-site datasets, emerging evidence suggests that DTI-derived metrics may improve early progression detection when used in conjunction with volumetric measures. However, adoption of diffusion MRI metrics as primary endpoints remains constrained by sensitivities to motion artifacts and the lack of cross-site harmonization in acquisition protocols.

#### Emerging and exploratory MRI biomarkers

Several additional MRI techniques are under investigation as exploratory biomarkers of PSP progression. Quantitative susceptibility mapping (QSM) has identified region-specific increases in iron deposition in the red nucleus, substantia nigra, and globus pallidus in PSP, although longitudinal validation remains limited (Langkammer et al., 2016). Neuromelanin-sensitive MRI enables visualization of neuromelanin-containing nuclei, including the substantia nigra and locus coeruleus, and has been explored in parkinsonian disorders as a marker of brainstem involvement (Cassidy et al., 2019). While preliminary studies suggest potential relevance in differentiating PSP from PD, longitudinal sensitivity and clinical correlations remain insufficiently characterized, necessitating further investigation (Marotta et al., 2024). Furthermore, resting-state functional MRI (fMRI) has revealed progressive disruption in functional connectivity within midbrain-thalamo-cortical networks, correlating with PSP variants (Sintini et al., 2024). In a recent study, PSP-RS showed widespread functional connectivity loss across the entire PSP network (cerebellum, midbrain, and cortex) while cortical variants primarily showed disruptions between the frontal cortex and thalamus, and subcortical variants showed more localized abnormalities in the basal ganglia (Sintini et al., 2024). However, the adoption of resting state fMRI as a progression biomarker is currently hindered by low signal-to-noise ratios and the lack of standardized analytical pipelines (Whitwell et al., 2011). Machine learning approaches integrating resting-state fMRI features, including structural, volumetric, and diffusion measures, are emerging, with the goal of improving sensitivity to progression and accommodating phenotypic heterogeneity (Cheng et al., 2025). While promising, these approaches require further validation, increased patient enrollment, and standardization before clinical trial deployment (Mattia et al., 2025).

#### Tau positron emission tomography

While MRI captures downstream neurodegeneration, PET aims to directly visualize the underlying proteinopathy *in vivo*. The temporal evolution of 4R tau pathology is thought to precede the clinical onset of PSP, necessitating the development of high-affinity in vivo imaging biomarkers. While second-generation tau PET radioligands show promise, PSP presents unique obstacles such as the subcortical density of tau is markedly lower than the cortical burden seen in AD (Dugger et al., 2011; Cope et al., 2018; Zhao et al., 2022), and the distinct folds of PSP-associated tau filaments often result in reduced binding affinity for traditional radioligands (Shi et al., 2021). Furthermore, as mentioned previously, inter-individual variability across PSP phenotypes (Table 1) may require nominated tau PET radioligands to map nuanced regional distribution patterns in the basal ganglia and brainstem. Despite these obstacles, the successful validation of 4R-specific radioligands would represent a significant neuroimaging advancement. Such biomarkers could enable earlier differential diagnosis, precise patient stratification for disease-modifying trials, and the objective monitoring of therapeutic efficacy by tracking longitudinal changes in subcortical tau deposition.

There are no FDA-approved tau PET radioligands for PSP. Current FDA-approved tau radioligand options remain limited to flortaucipir (18F-AV-1451, Tauvid™), which is indicated solely for AD. While flortaucipir is widely utilized in clinical research, currently appearing in 69 registered trials (Supplementary Figure 1), it exhibits significant off-target binding and poor affinity for the 4R tau filament structures characteristic of PSP (Schonhaut et al., 2017; Whitwell et al., 2020; Burnham et al., 2023; Satoh et al., 2024). Similarly, other investigational radioligands such as 18F-GTP1 and 18F-RO948 have demonstrated utility primarily in AD, with limited sensitivity for the 4R tauopathies (Leuzy et al., 2019; Cassinelli Petersen et al., 2022; Malarte et al., 2023). Although preliminary data for RO948 suggested potential utility in specific *MAPT* R406W mutation carriers (Santillo et al., 2023), its primary development remains focused on AD and amyloid-positive cohorts. 18F-MK-6240, another high-affinity ligand for AD tau, has recently shown reduced binding potency for non-AD isoforms, limiting its application in PSP (Gérard et al., 2025; Ghatamaneni et al., 2025).

To address the unmet need in PSP, second-generation radioligands have been engineered to improve binding characteristics for 4R tau aggregates. 18F-PI-2620 has emerged as a leading candidate for PSP imaging, demonstrating high-affinity *in vitro* binding to tau fibrils derived from both AD and primary tauopathies (Brendel et al., 2020; Mueller et al., 2020; Malarte et al., 2023; Young et al., 2024). Its clinical utility is currently being evaluated in 18 registered trials (Supplementary Figure 1). In 2024, the FDA granted Fast Track Designation to PI-2620 specifically for PSP, CBD, and AD indications, acknowledging its potential to fill a critical diagnostic gap (Hahn, 2024). Of note, a key phase 3 open-label multicenter trial (NCT05641688) is currently evaluating the efficacy of PI-2620 by correlating PET imaging with post-mortem histopathology. Furthermore, it is being utilized to assess tau network topology in atypical Parkinsonism patients within the University of Pennsylvania Centralized Observational Research Repository on Neurodegenerative Disease (UNICORN) initiative (NCT05456503). Although early clinical studies have demonstrated encouraging uptake patterns in PSP, the specificity of PI-2620 for PSP-associated 4R tau pathology and its relationship to regional tau burden remain under investigation. Ongoing clinicopathological studies, including postmortem correlation, will be important for establishing biological validity.

Structurally derived from the PBB3 scaffold (Maruyama et al., 2013), 18F-APN-1607 has shown potential for visualizing 4R tau in PSP (Li et al., 2021). Following the completion of a phase 1 longitudinal study in PSP patients (NCT05005819), the FDA granted APN-1607 Fast-Track Designation for the diagnosis of PSP in May 2024 (Meglio, 2024). This radioligand is currently under investigation in several trials, including observational studies for PSP in Taiwan (NCT04541836) and a combined PSP/FTD study in China (NCT05260151). These larger multicenter studies are needed to establish its reproducibility and diagnostic performance across the spectrum of PSP phenotypes.

The most recent entrant to the field is 18F-OXD-2314, a “pan-tau” ligand. In preclinical models, the parent compound, 18F-CBD-2115, demonstrated high affinity for all tau isoforms (3R, 4R, and mixed 3R/4R) (Lindberg et al., 2021). Later, OXD-2314 significantly improved brain uptake compared to its predecessor (Lindberg et al., 2024). First-in-human trials with a specific indication for PSP were initiated in Canada in late 2024 (Control number 285091), marking the start of its clinical phase (Murrell et al., 2025). As a first-in-human radioligand, its clinical performance, specificity for primary tauopathies, and potential off-target binding profiles have yet to be established.

As with all radiopharmaceuticals in the United States, the clinical translation of these ligands is overseen by the FDA, requiring adherence to Good Manufacturing Practice and rigorous safety evaluations parallel to standard drug development pipelines. The recent Fast Track designations for both PI-2620 and APN-1607 signal a regulatory recognition of the urgent need for validated neuroimaging biomarkers in the management of primary tauopathies like PSP. However, these designations should not be interpreted as evidence of clinical validity or diagnostic performance. Continued multicenter studies incorporating standardized imaging protocols and neuropathological confirmation will be critical to establish 4R-tau specificity, quantify residual off-target binding, and determine the reproducibility of these radioligands as biomarkers for clinical trials.

#### Fluorodeoxyglucose positron emission tomography

Among available neuroimaging tools, fluorodeoxyglucose PET (FDG-PET) has emerged as a robust metabolic biomarker used to measure regional cerebral glucose metabolism and therefore, inform the clinician on the synaptic activity and neuronal integrity of specific brain regions (Giannakis et al., 2025). Both interventional clinical trials for PSP that used PET as an outcome measure utilized the FDG-PET approach (NCT04184063 and NCT06162013). The diagnostic utility of FDG-PET in PSP stems from its ability to detect regional cerebral glucose hypometabolism, which often precedes structural atrophy visible on MRI (Beyer et al., 2018). There have been several studies demonstrating success in the supportive role of FDG-PET with the differential diagnosis of PSP variants from idiopathic PD and other parkinsonian syndromes (Reimold et al., 2011; Brajkovic et al., 2017; Buchert et al., 2025). In PSP-RS patients the characteristic metabolic signature includes significant hypometabolism in the midbrain and brainstem (often the earliest site of metabolic decline), basal ganglia (specifically, the striatum and caudate nucleus), the thalamus (closely linked to gait instability and falls) and frontal cortex (particularly the anterior cingulate and frontal eye fields, correlating with dysexecutive symptoms and vertical gaze palsy) (Seiffert et al., 2022; Strobel et al., 2023; Ali et al., 2024). By confirming a 4R-tauopathy metabolic pattern, clinicians may better select candidates for subsequent tau PET scans (Beyer et al., 2018).

Recent studies utilized non-negative matrix factorization on patients to identify distinct patterns of brain atrophy with MRI and hypometabolism with FDG-PET across the PSP spectrum (Ali et al., 2024). A key finding was that FDG-PET was a superior predictor of cortical features, such as limb apraxia and frontal dysexecutive syndrome, whereas MRI more accurately predicted subcortical symptoms like Parkinsonism. The study demonstrated that these imaging signatures are more robust at identifying the presence of a clinical symptom rather than its severity, suggesting they reflect the disease state more effectively than its stage. By combining modalities, the detection of complex phenotypes was enhanced highlighting that PSP-related neurodegeneration affects diverse motor, cognitive, and ocular networks through distinct anatomical foci.

#### 18 kDa translocator protein PET

PET imaging targeting the 18-kDa translocator protein (TSPO), a protein upregulated in the outer mitochondrial membrane of activated microglia, has become a tool in characterizing the neuroinflammatory state of patients with a variety of central nervous system (CNS) disorders (De Picker et al., 2023). Post-mortem studies have demonstrated dense microglial activation in the subcortical structures most affected by the disease (Ishizawa and Dickson, 2001). TSPO PET imaging provides an opportunity to examine this process in patients.

Early studies utilizing the first-generation ligand 11C-(R)-PK11195 demonstrated that PSP patients exhibit significantly higher binding in the basal ganglia and brainstem compared to healthy controls (Gerhard et al., 2006). Future studies showed that neuroinflammation is not merely a secondary feature of PSP but a driver of its clinical trajectory by demonstrating baseline TSPO-PET signal intensity in PSP targeted regions correlated to the rate of future clinical decline as measured by the PSPRS (Malpetti et al., 2021). Second-generation TSPO radioligands like 18F-GE-180 have been introduced (Vettermann et al., 2021), and efforts have focused toward harmonizing the data across many centers and methodologies to compare across PSP cohorts (Crook et al., 2026).

#### Synaptic vesicle glycoprotein 2A PET

Structural neuroimaging changes often fail to fully explain the severity of functional deficits early in the disease. Recent advancements have introduced radioligands targeting synaptic vesicle glycoprotein 2A (SV2A) which allow for the direct *in vivo* quantification of synaptic density to assess both disease severity and longitudinal progression (Bavarsad and Grinberg, 2024). Synaptic dysfunction is an early and prominent pathological event in tauopathies, frequently preceding macroscopic atrophy, and cross-sectional PET studies utilizing 11C-UCB-J have demonstrated reductions in synaptic density in patients with PSP-RS (Holland et al., 2020, 2022). Compared to age-matched controls, PSP patients exhibited significant decreases in SV2A binding across cortical and subcortical structures, a finding that was also shared in a study of patients with behavior variant FTD (bvFTD) (Carson et al., 2022; Malpetti et al., 2023; Visser et al., 2024).

The interpretation of in vivo SV2A PET data in PSP has been bolstered by post-mortem validation which confirmed that in vivo 11C-UCB-J binding corresponds to the expression of SV2A and other presynaptic markers like synaptophysin in PSP brain tissues (Scarpa et al., 2025). This supports SV2A PET as a proxy for total synaptic integrity in 4R tauopathies. Furthermore, recent studies have identified significant reductions in SV2A density in PSP patients across most examined regions, with the frontal cortices being the most severely impacted (Shanaki Bavarsad et al., 2024). While PSP exhibited significant synaptic loss, the magnitude of depletion in cortical areas was generally less severe than that observed in early-onset AD or FTLD-GRN. Because frontal synaptic loss was observed, SV2A imaging may serve as a sensitive metric for monitoring disease progression and assessing the efficacy of future neuroprotective therapies in clinical trials.

### Fluid-based biomarkers

**Figure 3:**
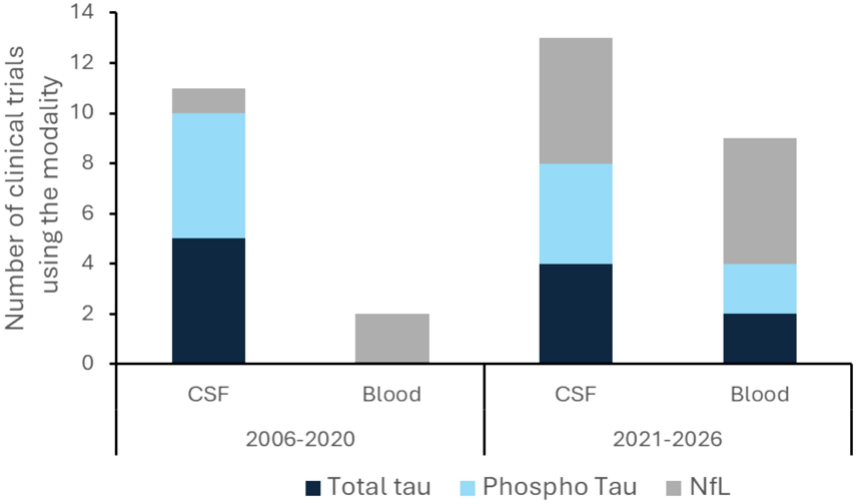
Utilization of fluid biomarkers to support outcome measurements in PSP clinical trials initiated during the referred time interval.

Fluid-based biomarkers are becoming increasingly utilized in PSP interventional trials (Figure 3). Of the 13 clinical trials initiated since 2021, seven have utilized fluid biomarkers to support outcome assessments. With the improvement in method development and diagnostic platform sensitivity and selectivity, there is a growing utilization of fluid measurements to accompany other outcome measures.

#### Neurofilament light

Neurofilament light chain (NfL) is a structural protein in myelinated axons (Coppens et al., 2023). Following axonal injury, NfL is released into the cerebrospinal fluid (CSF) and subsequently into the bloodstream. Both CSF and blood (serum or plasma) NfL concentrations are elevated in PSP, and higher NfL levels are associated with greater functional, motor, and cognitive deficits, a worse survival rate, and severity of disease and progression indicating its potential use as a monitoring and prognostic biomarker in PSP (Rojas et al., 2016, 2018; Donker Kaat et al., 2018; Constantinescu et al., 2019; Coppens et al., 2023). However, NfL has shown limited utility as a diagnostic biomarker for PSP due to its lack of specificity. Comparative studies show that NfL levels are elevated across multiple neurodegenerative diseases limiting NfL’s use as a standalone diagnostic biomarker (Wang et al., 2019; Delaby et al., 2020; Bendstrup et al., 2022). That said, one potential use of NfL is supporting the differential diagnosis of PSP from PD, a common misdiagnosis for patients due to early clinical symptom overlap. Results have shown that NfL levels are lower in PD than PSP and using NfL levels alongside other biomarkers and clinical presentation leads to more accurate differentiation of PSP from PD (Sako et al., 2015; Marques et al., 2019; Mangesius et al., 2020; Li et al., 2026).

Numerous studies demonstrate that NfL’s use as a biomarker for PSP is improved in combination with other available molecular and imaging biomarkers. Plasma NfL concentrations correlate with structural MRI changes, linking NfL levels to neurodegenerative burden (Boxer et al., 2014). Measuring both p-tau 181 and NfL in the CSF of PSP patients correlated with disease severity and rate of progression, and the ratio of NfL to p-tau was better at predicting changes in the PSPRS clinical rating scale, than either measure on its own (Rojas et al., 2018). Combining imaging measures of regional atrophy with NfL measures showed that higher levels of localized inflammation in subcortical regions and plasma NfL measures were correlated with shorter survival, demonstrating that complementary markers may be more sensitive when combined than other measures alone and improved over clinical rating scales alone (Shapiro et al., 2025). Taken together, these findings suggest that NfL’s utility as a biomarker in PSP is increased when used in with complementary biomarkers, and that this multimodal biomarker framework has potential to perform better than traditional clinical rating scales.

In summary, NfL shows promise as a monitoring or prognostic biomarker in PSP drug development, particularly when used alongside other available biomarkers and clinical measures. One advantage of NfL as a biomarker is its accessibility. It can be measured in CSF or blood with several measurement assays providing consistent NfL values in plasma and CSF (Sheth et al., 2026). NfL has been incorporated into past PSP therapeutic trials, and is included in ongoing longitudinal studies such as ALLFTD aimed at further characterizing biomarkers in PSP (Boxer et al., 2014; Höglinger et al., 2021; Sheth et al., 2025). As biomarker research in PSP advances, recent work has proposed that NfL could contribute to multimodal panels of several biomarkers which could define biologically distinct subgroups of PSP which would allow for improved patient stratification in future clinical trials (Lee et al., 2026; Ryan et al., 2026).

#### Tau-based

CSF remains the most proximal biofluid to the CNS, offering a direct window into brain-derived tau species. In AD, the levels of both CSF total tau (t-tau) and p-tau at residue 181 (p-tau181) are elevated (Jack et al., 2018). The first longitudinal study of t-tau and p-tau181 in a PSP clinical cohort occurred in the davunetide trial (NCT01110720) where no significant differences in either tau measure between the group receiving davunetide and the treatment and placebo groups over the 52-week period was observed (Boxer et al., 2014). Subsequent studies monitoring CSF t-tau and p-tau181 levels have shown similar levels between patients with the clinical diagnosis of PSP and controls (Ishiguro and Kasuga, 2024). Additionally, the antemortem levels of both t-tau and p-tau181 were found to be decreased in autopsy-confirmed PSP cases (Mattsson-Carlgren et al., 2022). Many hypotheses have been proposed for these observations (Ishiguro and Kasuga, 2024), but no explanation has been demonstrated experimentally. As a result, traditional AD CSF tau biomarkers have demonstrated limited use for PSP patients.

The landscape of tau has been recently dominated by p-tau217, a variant p-tau that has shown to be an exceptionally accurate surrogate for AD pathology (Ashton et al., 2023; Khalafi et al., 2025; Palmqvist et al., 2025). However, for patients of PSP, p-tau217 serves a paradoxical role in that it is valuable not for what it detects, but for what it fails to find. Studies have demonstrated that while p-tau217 levels are high in AD, they remain notably low in patients with PSP (Thijssen et al., 2021; VandeVrede et al., 2023; Benussi et al., 2025). A theory that may explain this phenomenon involves tau isoform specificity and sequestration. Unlike the mixed 3R/4R tau aggregates found in AD, PSP is characterized by 4R tau. Standard p-tau217 assays were largely optimized to detect the soluble tau fragments associated with amyloid-beta plaques found in AD. In PSP, the tau protein is believed to be tightly sequestered within insoluble neurofibrillary tangles and tufted astrocytes, preventing its release into the CSF or blood. Therefore, a patient with severe midbrain atrophy may present with a normal phosphorylated tau profile (Boxer et al., 2017). Finally, recent extensive tissue proteomic studies have revealed lower overall abundance of insoluble tau, cleavage sites, and post-translational modifications including phosphorylation sites associated with PSP samples compared to other tauopathies (Kumar et al., 2026). Given a low abundance of both insoluble and modified tau species detected within PSP brain tissue, it follows that the levels found in soluble forms would also be low.

The most significant development recently has been the focus on the microtubule-binding region (MTBR) of tau. While standard t-tau assays primarily measure N-terminal or mid-domain fragments, the MTBR contains the core of the insoluble filaments that define PSP. Like other N-terminal or mid-domain tau fragments (e.g., p-tau181), the reports of CSF tau fragments containing the MTBR, specifically those starting at residue 243 (MTBR-tau243) were significantly elevated in AD and frontotemporal lobar degeneration (FTLD) *MAPT* R403W mutation carriers, and not 4R tauopathies like PSP (Horie et al., 2023; Horie et al., 2025a). However, alternative MTBR fragments containing the R2 repeat region (MTBR-tau275 and MTBR-tau282) were uniquely altered in PSP and CBD (Horie et al., 2022). While both are 4R tauopathies, the specific enrichment of both CSF MTBR-tau275/t-tau and CSF MTBR-tau282/t-tau in CBD vs. PSP (with and without AD pathology present) yielded a Receiver Operating Characteristic (ROC) Area Under the Curve (AUC) of greater than 0.82. Current research on MTBR tau is focusing on whether the MTBR-tau fragments identified in CSF can be detected in plasma. Early data suggests that the ratio of endogenous plasma MTBR-tau243 can help differentiate 4R tauopathies from 3R/4R mixed tauopathies like AD (Horie et al., 2025b).

The recent discovery of assays focused on brain-derived tau (BD-tau) has revealed another approach to correlate blood-based biomarkers from proteins that originated in the CNS (Gonzalez-Ortiz et al., 2023). The tau in the brain has a unique signature corresponding to how it is spliced and assembled. In peripheral tau (often referred to as Big Tau), the *MAPT* gene encodes exon 4a whereas in BD-tau, it is not included. Many routinely used total-tau antibodies do not differentiate between the types of tau adequately, but the antibodies used for BD-tau recognize the contiguous sequence of exons 4–5 which affords additional specificity against Big Tau fragments. Unlike traditional total-tau assays, BD-tau levels in the blood show a high correlation with CSF markers and are uniquely sensitive to AD-specific neurodegeneration (Gonzalez-Ortiz et al., 2024). Data confirming its validity to primary tauopathies, especially PSP, is limited. It has shown much weaker utility possibly due to the lack of amyloid-beta pathology or blood–brain barrier breakdown which may release more BD-tau into the bloodstream in some neurodegenerative conditions than others (Gonzalez-Ortiz et al., 2023; Marotta et al., 2025). Therefore, there is a clear need to refine the use of BD-tau assays in future blood-based biomarker studies of primary tauopathy samples.

#### Seed amplification assays

Seed amplification assays (SAAs) have emerged as a molecular approach for detecting misfolded protein aggregates in neurodegenerative diseases. These assays exploit the prion-like seeding properties of pathogenic proteins, in which small amounts of misfolded aggregates present in biological samples template the conversion of recombinant substrate proteins into amyloid fibrils under controlled conditions. The resulting amplification reaction generates a detectable fluorescence signal, allowing extremely low concentrations of pathogenic seeds to be identified in biological samples such as CSF, skin, or other peripheral tissues (Candelise et al., 2020; Vascellari et al., 2022). Initially developed for prion diseases, SAAs have demonstrated excellent diagnostic performance for sporadic Creutzfeldt–Jakob disease and represent a major advance in the biomarker-based detection of protein misfolding disorders (Groveman et al., 2017).

The translational potential of SAAs has become evident with the development of *α*-synuclein SAAs, which detect pathological aggregates associated with synucleinopathies such as PD, dementia with Lewy bodies (DLB), and multiple system atrophy (MSA). Multiple studies have demonstrated that α-synuclein SAAs can identify seeding activity in CSF with high sensitivity and specificity, and pathological seeds have also been detected in peripheral tissues including olfactory mucosa and skin biopsies (Rossi et al., 2020; Nakagaki et al., 2021; Coysh and Mead, 2022). Importantly, these assays have shown the ability to distinguish between different synucleinopathies, including PD and MSA, which exhibit distinct conformational strains of α-synuclein aggregates (Poggiolini et al., 2022). As a result, α-synuclein SAAs are increasingly being incorporated into biomarker frameworks aimed at identifying patients with underlying synuclein pathology. The successful translation of α-synuclein SAAs into clinical research settings therefore provides compelling proof-of-concept that seed amplification technologies can move beyond experimental applications and serve as clinically meaningful diagnostic biomarkers.

Building on this precedent, there is growing interest in adapting SAAs to investigate tauopathies. In these disorders, pathological tau aggregates exhibit disease-specific conformations and propagation properties that likely reflect distinct molecular “strains” of tau (Stamelou et al., 2021; Koga et al., 2022). Seed amplification technologies offer a potential strategy to detect these pathogenic tau assemblies and capture disease-specific molecular signatures. Experimental studies using SAA platforms have demonstrated that 4R-tau seeds derived from PSP and CBD brain tissue can be amplified *in vitro*, suggesting that these assays may help distinguish overlapping tauopathies and provide insights into disease biology. Recent work further highlights the potential relevance of this approach for understanding PSP heterogeneity. Using a 4R-tau seed amplification assay applied to postmortem PSP brain tissue, Martinez-Valbuena and colleagues demonstrated substantial interindividual variability in tau seeding activity that correlated with biochemical features of high-molecular-weight tau assemblies and distinct molecular signatures (Martinez-Valbuena et al., 2025). These findings suggest that tau seeding capacity may reflect underlying molecular subtypes of PSP, raising the possibility that SAA-based approaches could ultimately help stratify patients for clinical trials. Additionally, recent work presented a minimally invasive diagnostic strategy that integrates dermal α-synuclein and 4R tau SAAs from a single skin biopsy alongside serum NfL measurements to differentiate neurodegenerative parkinsonian syndromes (Martinez-Valbuena et al., 2026). By combining these complementary biomarkers, the researchers successfully bypassed the limitations of single-protein tracking to accurately distinguish between PD, MSA, and PSP. The approach also captured biological heterogeneity by successfully identifying a subset of PSP patients with underlying α-synuclein co-pathology.

Despite these promising developments, several challenges remain before SAAs can be widely implemented in clinical practice. Variability in protocols, including differences in recombinant substrates, reaction conditions, and sample preparation, can influence SAA sensitivity and aggregation kinetics, complicating comparisons across laboratories and limiting standardization (Dong and Satoh, 2021; Vascellari et al., 2022). In addition, factors including sample collection, handling, storage, and freeze–thaw conditions, may further influence assay performance and require standardized protocols before widespread clinical adoption. While α-synuclein SAAs have shown reproducibility across independent cohorts, similar validation efforts will be necessary for tau-based SAAs. Large multicenter replication studies and methodological harmonization will therefore be essential to establish robust assay performance, define clinically meaningful thresholds, and determine how seeding activity relates to disease stage and progression. Furthermore, prospective longitudinal studies are needed to determine the stability of tau seeding activity over time and to establish whether SAAs can reliably monitor disease progression, predict clinical outcomes, or serve as biomarkers in therapeutic trials.

#### Other emerging protein and molecular biomarkers

In addition to tau-focused approaches, a growing body of research is exploring inflammatory and multi-omic biomarkers to further refine disease characterization. Astrocytic activation markers, particularly glial fibrillary acidic protein (GFAP), have emerged as potential blood-based biomarkers for PSP (Sheth et al., 2025). Plasma GFAP levels are significantly elevated in PSP and show a strong correlation with clinical severity and longitudinal atrophy of the brainstem and striatum (Chen et al., 2025). However, studies spanning atypical parkinsonian syndromes indicate substantial overlap in GFAP elevations across neurodegenerative diseases, suggesting that GFAP is most informative when incorporated into multi-marker panels rather than used as a standalone progression biomarker (Campagnolo et al., 2025).

Additional neuroinflammatory markers are under investigation, including soluble Triggering Receptor Expressed on Myeloid Cells 2 (sTREM2), a marker of microglial activation that is elevated across multiple neurodegenerative disorders. Preliminary evidence indicates that sTREM2 mRNA expression is upregulated in the substantia nigra of patients with PSP, although data on sTREM2 levels in the blood and the clinical implications for PSP remain limited (Lee et al., 2026). Emerging multi-omic studies have also identified immune-related markers such as complement component 4a (C4A) and human cartilage glycoprotein 39 (YKL-40) as potential markers that distinguish PSP from related disorders and correlate with rapid functional decline, although these findings require further validation (Farrell et al., 2024).

Recent research underscores the pivotal role of neuroinflammation and peripheral immune dysregulation in the pathogenesis and clinical progression of PSP. There have been efforts to identify a pro-inflammatory blood-based profile across the FTLD spectrum, including PSP, that correlates with central neuroinflammation and served as a negative predictor of survival, independent of baseline clinical severity (Malpetti et al., 2025). This is supported by 11C-PK11195 PET imaging of the neuroinflammation target TSPO, which demonstrated localized subcortical inflammation, in conjunction with elevated plasma NfL levels, providing a prognostic framework for predicting shorter survival in PSP patients (Shapiro et al., 2025). Complementing these longitudinal insights, high-sensitivity proteomic panels revealed that PSP is characterized by the upregulation of specific inflammatory markers, such as matrix metalloproteinase-9 (MMP9) and hepatocyte growth factor (HGF), distinguishing it from AD and nominating immune signatures as potential diagnostic predictors of disease outcomes (Durcan et al., 2025). Collectively, these studies suggest that both systemic and regional inflammatory biomarkers may improve diagnostic accuracy, monitoring disease progression, and identifying potential therapeutic targets in PSP.

While PSP is primarily a sporadic disorder, genomic markers are increasingly used to predict the rate of progression and phenotype. The *MAPT* H1 haplotype remains the most significant genetic risk factor, however, recent evidence highlights that specific subhaplotypes, such as H1c, are associated with more aggressive clinical courses and higher tau burden (Farrell et al., 2024; Ressler et al., 2024). Furthermore, variants in the leucine-rich repeat kinase 2 (LRRK2) gene, which are traditionally associated with PD, have been identified as key determinants of progression speed in PSP. Patients carrying specific LRRK2 variants exhibit a significantly slower rate of motor decline, offering a potential genetic stratification tool for clinical trials (Buck et al., 2025; Müller et al., 2025; Nielsen et al., 2025). Other risk loci, including myelin-associated oligodendrocyte basic protein (MOBP) and syntaxin-6 (STX6), are now being integrated into polygenic risk scores to refine prognostic modeling and survival prediction in PSP (Farrell et al., 2024).

### Digital health technologies and electrophysiological approaches

Because PSP is characterized by early postural instability and frequent falls (Höglinger et al., 2017), movement and gait-based measures derived from wearable sensors have emerged as a disease-relevant category of digital health technologies for PSP biomarker development (Isroff et al., 2025). Recent longitudinal studies indicate that these tools can detect statistically significant disease progression within intervals as short as three to 6 months, outperforming the sensitivity of traditional clinical scales (Sotirakis et al., 2022). Specifically, metrics such as turning velocity and step-duration variability have been shown to mirror the rate of progressive midbrain and brainstem atrophy, providing a direct link between digital motor outputs and underlying neurodegeneration (Abate et al., 2023).

Gait analysis studies examining short-step and wide-based gaits, as well as measures of gait velocity and dynamic instability, have demonstrated high utility for both early-stage screening and as longitudinal trial endpoints for monitoring PSP progression in clinical trials (Takamatsu et al., 2019; Amboni et al., 2021). By enabling continuous, remote monitoring in naturalistic environments, scalable sensors can offer a robust framework for tracking disease severity and evaluating therapeutic efficacy (Abate et al., 2023; Sharma et al., 2023). While digital health technologies are widely used as exploratory measures, they are increasingly being incorporated as biomarkers and are currently utilized as outcomes in five PSP interventional trials (NCT042222180, NCT04237948, NCT04608604, NCT04655079, and NCT06162013). Furthermore, these technologies are undergoing rigorous validation in formal prospective studies, such as NCT07389018. In these settings, integrated digital endpoints provide objective, high-fidelity, and low-burden metrics of disease progression that serve as complements to traditional clinician-reported scales (Isroff et al., 2025).

Oculomotor dysfunction is a defining feature of PSP and therefore represents a highly disease-specific substrate for digital biomarker development. Eye-tracking studies consistently demonstrate impairments in vertical saccade velocity, latency, and gaze range that reflect degeneration of midbrain oculomotor nuclei and correlate with clinical severity (Garbutt, 2004; Marx et al., 2012). Importantly, it has been shown that quantitative eye-tracking metrics can distinguish PSP from *α*-synucleinopathies, identifying oculomotor signatures that differentiate tau-mediated from synuclein-mediated neurodegeneration and supporting their diagnostic and stratification utility (Habibi et al., 2022). More recently, reports have highlighted eye tracking as a scalable digital biomarker across neurodegenerative diseases, including PSP, emphasizing its sensitivity to longitudinal change, low patient burden, and suitability for remote or clinic-based monitoring (Giacomini et al., 2026). Building on these disease-specific digital approaches, emerging work in related neurodegenerative syndromes such as FTD has demonstrated the feasibility of smartphone and app-based digital assessments for capturing cognitive and motor dysfunction in real-world settings, suggesting that similar platforms could be adapted and validated for PSP to extend continuous monitoring beyond gait and oculomotor measures (Taylor et al., 2023; Staffaroni et al., 2024).

Emerging work also supports the potential role of speech-based digital measures in PSP. Quantitative speech analyses capturing features such as articulation, speech timing, prosody, and acoustic variability may provide scalable and remotely deployable measures of motor and cognitive dysfunction relevant to PSP progression (Kang et al., 2023; Di Rauso et al., 2025). Recent studies utilizing automated speech analysis and machine learning approaches further suggest that speech-derived measures may aid in differentiating PSP from related neurodegenerative disorders while enabling sensitive, low-burden longitudinal monitoring (Kang et al., 2025). While still at an earlier stage of validation compared with gait and oculomotor approaches, speech-based biomarkers represent a promising emerging modality that could contribute to multidimensional disease monitoring frameworks and future biomarker development efforts in PSP.

Complementing digital measures of motor, oculomotor, and speech dysfunction, electroencephalography (EEG) provides noninvasive measures of large-scale network dysfunction in PSP and complements structural and fluid biomarkers by capturing functional consequences of tau-mediated neurodegeneration (Mostile et al., 2024). Quantitative EEG (qEEG) analyses further show that spectral power shifts and altered coherence measures correlate with cognitive impairment and overall disease severity, supporting their potential utility as progression-sensitive biomarkers (Colom-Cadena et al., 2020; Liu et al., 2023). Importantly, neurophysiological studies directly comparing PSP with PD patients demonstrated distinct EEG signatures in PSP, indicating that these network abnormalities may reflect disease-specific tau-related dysfunction rather than nonspecific Parkinsonism (Liu et al., 2023).

Despite these advances, broader implementation of digital biomarkers will require standardized acquisition protocols, harmonized analytical pipelines, and validation across diverse clinical populations to ensure reproducibility and interoperability between devices and study sites. Looking forward, the integration of digital biomarkers with neuroimaging, fluid biomarkers, and clinical measures using artificial intelligence and multimodal computational frameworks may enable more precise patient stratification, individualized disease monitoring, and biomarker-guided therapeutic development. Together, digital motor, oculomotor, speech-based, app-based, and electrophysiological biomarkers provide complementary, scalable tools for capturing functional decline in PSP and represent an important component of future precision medicine approaches and multidimensional clinical trial frameworks.

## Conclusion

The evolution of PSP research has reached a critical inflection point, moving from a reliance on clinical phenotypes toward a paradigm of molecular precision. While the 1963 characterization of Richardson’s Syndrome remains foundational, the discovery of diverse clinical variants and the unique three-layered fold of 4R-tau filaments have necessitated more sophisticated biomarker tools. Traditional clinical rating scales, though useful for measuring disease burden, often lack the sensitivity and inter-evaluator reliability required for modern clinical trials. Consequently, the integration of neuroimaging and fluid biomarkers is essential to delineate PSP from overlapping syndromes.

Advanced neuroimaging offers a direct window into the disease’s structural and metabolic progression. While volumetric MRI and the MRPI 2.0 provide robust measures of subcortical atrophy, the emergence of second-generation tau PET ligands like 18F-PI-2620 and 18F-APN-1607, both of which have received FDA Fast Track designation, and the pan-tau radioligand OXD-2314 signal a breakthrough in our ability to visualize 4R-tau pathology *in vivo*. Furthermore, metabolic markers like FDG-PET and new radioligands for SV2A and neuroinflammation with TSPO provide complementary data that can better reflect the functional state of the brain than structural imaging alone.

Parallel advancements in fluid biomarkers have enhanced our capacity for prognostic monitoring and differential diagnosis. Although markers like p-tau217 are invaluable for identifying AD, their characteristically low levels in PSP support a negative diagnostic signature that prevents misdiagnosis. The discovery of specific MTBR tau fragments, such as MTBR-tau275 and MTBR-tau282, offers a promising path toward distinguishing PSP from other 4R tauopathies, but more studies need to be conducted to confirm. Additionally, the widespread accessibility of NfL provides a practical, albeit nonspecific means of tracking neurodegenerative burden and predicting clinical trajectory.

Ultimately, the most effective strategy for unlocking the 4R-tau therapeutic pipeline and setting the stage for more impactful clinical trials lies in a multimodal biomarker framework. Emerging approaches, including seed amplification assays and digital biomarkers, further extend this framework by enabling molecular-level disease stratification and high-frequency, real-world monitoring of disease progression, respectively. By combining the anatomical specificity of imaging with the molecular sensitivity of fluid assays, researchers can improve patient selection, enable adaptive platform trials, and provide objective measures of target engagement. Rather than relying on any single biomarker, future multimodal assessment frameworks will likely combine structural, molecular, functional, and digital biomarkers into composite models that more accurately reflect the biological heterogeneity of PSP and support precision medicine approaches. This shift toward biomarker-enabled precision has the potential to transform PSP from a condition diagnosed largely at autopsy into one that can be more accurately staged and treated in the earliest phases of clinical presentation.
