# Supplementary material for: From clinical phenotypes to molecular precision: multimodal biomarkers for progressive supranuclear palsy
Source: Front Neurosci. 2026 Jul 27;20:1893149. doi: 10.3389/fnins.2026.1893149 (PMC13454329; doi:10.3389/fnins.2026.1893149)
Supplement: Supplementary file 1 [file Data_Sheet_1.docx]

Supplementary information

From Clinical Phenotypes to Molecular Precision: Multimodal Biomarkers for Progressive Supranuclear Palsy

Glenn A. Harris^1*^, Lymor Barnhard^1^, Jennifer Brummet^2^, Kristophe Diaz^2^

^1^ Rainwater Charitable Foundation, 777 Main Street, Suite 2250, Fort Worth, TX, 76102, USA

^2^ CurePSP, 325 Hudson Street, 4th Floor, New York, NY 10013, USA

*** Correspondence:**
Corresponding Author
gharris@rainwatercf.org

Keywords: PSP, biomarkers, neurodegeneration, neuroimaging, tauopathy, tau

**Supplementary Table 1: Therapeutic clinical trials targeting PSP since 2006 (indexed to April 1, 2026)**

| **NCT Number** | **Study Title** | **Phase** | **Start Date** |
| --- | --- | --- | --- |
| NCT00328874 | Study About Safety and Efficacy of Coenzyme Q10 in PSP | 2 | 2006-05 |
| NCT00382824 | Effects of Coenzyme Q10 in PSP | N/A | 2006-09 |
| NCT00385710 | Trial of Valproic Acid in Patients With PSP (Depakine) | 2 | 2006-11 |
| NCT00703677 | A Pilot Trial of Lithium in Subjects With PSP or CBD | 1, 2 | 2008-09 |
| NCT01049399 | Safety, Tolerability, and Efficacy of Two Different Oral Doses of NP031112 Versus Placebo in the Treatment of Patients With Mild-to-Moderate PSP | N/A | 2009-12 |
| NCT01056965 | Davunetide (AL-108) in Predicted Tauopathies - Pilot Study | 1 | 2010-01 |
| NCT01187888 | Efficacy, Tolerability and Safety of Azilect in Subjects With PSP | 3 | 2010-01 |
| NCT01537549 | Alpha-lipoic Acid/L-acetyl Carnitine for PSP | 1, 2 | 2010-09 |
| NCT01110720 | Study to Evaluate the Safety and Efficacy of Davunetide for the Treatment of PSP | 2, 3 | 2010-10 |
| NCT02133846 | Safety Study of TPI-287 to Treat CBS and PSP | 1 | 2014-05 |
| NCT02422485 | A 6 Month, Open-Label, Pilot Futility Clinical Trial of Oral Salsalate for PSP | 1 | 2015-04 |
| NCT02460731 | Young Plasma Transfusions for PSP | 1 | 2015-05 |
| NCT02658916 | Extension Study of BIIB092 in Participants With PSP Who Participated in CN002003 | 1 | 2016-02 |
| NCT02839642 | Efficacy of RIVAstigmine on Motor, Cognitive and Behavioural Impairment in PSP | 3 | 2016-07 |
| NCT03068468 | Study of BIIB092 in Participants With PSP | 2 | 2017-06 |
| NCT03391765 | An Extension Study of ABBV-8E12 in PSP | 2 | 2018-01 |
| NCT03413319 | Extension Study of ABBV-8E12 in Patients With PSP Who Completed Study C2N-8E12-WW-104 | 1 | 2018-04 |
| NCT04184063 | Study of NBMI Treatment in Patients With Atypical Parkinsons (PSP or MSA) | 2 | 2019-09 |
| NCT04185415 | A Study to Test the Safety and Tolerability of UCB0107 in Study Participants With PSP | 1 | 2019-12 |
| NCT04008355 | A Study to Assess Tolerability, Safety, Pharmacokinetics and Effect of AZP2006 in Patients With PSP | 2 | 2020-06 |
| NCT04658199 | A Study to Test the Safety and Tolerability of Long-term UCB0107 Administration in Study Participants With PSP | 1 | 2020-11 |
| NCT04253132 | Evaluation of Tolfenamic Acid in Individuals With PSP at 12-Weeks | 1, 2 | 2021-01 |
| NCT04734379 | Rho Kinase (ROCK) Inhibitor in Tauopathies – 1 | 2 | 2021-01 |
| NCT04539041 | Safety, Tolerability and Pharmacokinetics of Multiple Ascending Doses of NIO752 in PSP | 1 | 2021-02 |
| NCT04937530 | RT001 in Patients With PSP | 2 | 2021-06 |
| NCT04993768 | A Phase 2a Study of TPN-101 in Patients With PSP | 2 | 2021-10 |
| NCT05819658 | GV1001 Subcutaneous(SC) for the Treatment of PSP | 2 | 2023-06 |
| NCT06235775 | Administration of GV1001 for the Treatment of PSP Who Completed Study GV1001-PSP-CL2-011 | 2 | 2023-12 |
| NCT06122662 | AMX0035 and PSP | 2, 3 | 2023-12 |
| NCT06162013 | The NADAPT Study: a Randomized Double-blind Trial of NAD Replenishment Therapy for Atypical Parkinsonism | 2 | 2024-03 |
| NCT06355531 | A Study to Assess the Efficacy, Safety, and Pharmacokinetics of FNP-223 to Slow Progression of PSP | 2 | 2024-07 |
| NCT07264283 | The PSP Clinical Trial Platform - Regimen B: LM11A-31 | 2 | 2025-12 |
| NCT07217665 | The PSP Clinical Trial Platform - Regimen A: AADvac1 | 2 | 2025-12 |
| NCT07498426 | A Study to Evaluate the Efficacy of NIO752 in Participants With PSP | 3 | 2026-05 |

**Supplementary Figure 1:** Clinical trial landscape of 18F-Tau PET tracers appearing in Clinicaltrials.gov (indexed to Dec. 31, 2025)
